# Acute Myeloid Leukemia Affects Mouse Sperm Parameters, Spontaneous Acrosome Reaction, and Fertility Capacity

**DOI:** 10.3390/ijms20010219

**Published:** 2019-01-08

**Authors:** Yulia Michailov, Eitan Lunenfeld, Joseph Kapilushnik, Shevach Friedler, Eckart Meese, Mahmoud Huleihel

**Affiliations:** 1 The Shraga Segal Department of Microbiology, Immunology, and Genetics, The Center of Advanced Research and Education in Reproduction (CARER), Faculty of Health Sciences, Ben-Gurion University of the Negev, Beer-Sheva 8410501, Israel; yuliadiuro@gmail.com; 2 IVF Unit, Barzilai University Medical Center, Ashkelon 7830604, Israel; shevachf@bmc.gov.il; 3 The Center of Advanced Research and Education in Reproduction (CARER), Department OB/GYN, Soroka Medical Center and Faculty of Health Sciences, Ben-Gurion University of the Negev, Beer-Sheva 8410501, Israel; lunenfld@bgu.ac.il; 4 The Center of Advanced Research and Education in Reproduction (CARER), Faculty of Health Sciences, Ben-Gurion University of the Negev, Beer-Sheva 8410501, Israel; kapelush@bgu.ac.il; 5 Faculty of Health Sciences, Ben-Gurion University of the Negev, Beer-Sheva 8410501, Israel; 6 Department of OBGYN and Infertility, Barzilai University Medical Center, Ashkelon 7830604, Israel; 7 Institute of Human Genetics, Saarland University, Homburg/Saar, 66421 Homburg, Germany; eckart.meese@uks.eu

**Keywords:** acute myeloid leukemia, male infertility, sperm parameters, testis, acrosome reaction

## Abstract

Leukemia is one of the most common cancers in patients of reproductive age. It is well known that chemotherapy, used as anti-cancer therapy, adversely affects male fertility. Moreover, the negative effect of leukemia on sperm quality, even before chemotherapy treatment, has been reported. However, the mechanisms behind this disease’s effect on sperm quality remains unknown. In this study, we examine the direct effect of leukemia and chemotherapy alone and in combination on sperm parameters and male fertility. For this, we developed an acute myeloid leukemia (AML) mouse model (mice were treated with AML cells C1498 and developed leukemia); these mice then received cytarabine chemotherapy. Our findings reveal a significant reduction in sperm concentration and motility and a significant increase in abnormal morphology and spontaneous acrosome reaction of the sperm following AML and chemotherapy treatment, alone and in combination. We also found a reduction in male fertility and the number of delivered offspring. Our results support previous findings that AML impairs sperm parameters and show for the first time that AML increases spontaneous acrosome reaction and decreases male fertility capacity and number of offspring.

## 1. Introduction

Leukemia is one of the most common cancers of patients of reproductive age. Treatment of these patients with anticancer therapy (aggressive chemotherapy and/or radiotherapy) has known side effects on the reproductive system that may lead to permanent male sterility and, in a large proportion of patients, to oligospermia or azoospermia [1]. This effect is dependent on the type, cumulative dose, treatment duration, and potential interactions between various combination regimes. The damage effect of chemotherapy on spermatogenesis is often permanent, but in some patients, a few stem cells resist this destruction and survive the treatment, resulting in a recovery of fertility sometimes many years after the treatment ends, especially in those treated with chemotherapeutic agents with alkylating properties [2,3,4,5,6,7,8,9]. The only established method of preserving the potential of reproductive capacity in adult cancer patients is sperm cryopreservation before anti-cancer treatments [10]. The cryopreserved sperm can then be used in artificial reproductive techniques, usually in-vitro fertilization and intracytoplasmic sperm injection [10,11].

Recently, it was reported that sperm parameters from leukemia patients were impaired even before anti-cancer treatments [12,13,14,15]. Chung et al. (2004) showed the presence of oligozoospermia in 57% of leukemia patients [16]. Auger et al. (2016) found a significant decrease in sperm motility and morphology in most patients, and normal sperm parameters were visible only in 36.9% of them [17]. Moreover, lymphoid leukemia patients demonstrated a significant reduction in total motile counts and motility compared to other cancer patients, including those with testicular, brain, and Hodgkin’s lymphoma cancers [18]. In additional studies, leukemia patients had the lowest sperm motility (28.7%) compared to other examined cancer patients (testicular cancer, non-Hodgkin’s disease, Hodgkin’s disease, gastrointestinal malignancy, and musculoskeletal malignancy) (30.2%) [19]. A significant reduction in the motile sperm counts, sperm motility, and curve linear velocity compared to healthy donors was reported [20]. In addition, leukemia patients had a lower concentration and motility of sperm compared to patients with other cancers (testicular, brain, lymphoma, prostate, sarcoma, colorectal, unspecified, and others) [21]. Further studies report that sperm parameters (concentration, necrospermia rate, and atypical sperm) from chronic myeloid leukemia patients were impaired compared to a control [22]. Moreover, sperm parameters (survival rate, motile sperm count, motility, and curvilinear velocity) of thawed samples that were cryopreserved from leukemia patients were impaired compared to a control [18,20].

Systemic and local effects of a cancer can impact fertility, although the exact mechanism has not yet been clarified [23]. Leukemia may evoke a systemic response of the body. Cytokines such as interleukins, and tumor necrosis factors and other factors secreted by tumor cells and the immune system cells may mediate this systemic response [23]. Imbalance in testicular cytokines and growth factors may impair the process of spermatogenesis by affecting the proliferation and differentiation of spermatogonial stem cells (SSCs) and increase the apoptosis of spermatogenic cells, leading to infertility. In addition, the physical and emotional stress that accompanies a cancer diagnosis can impair semen quality through an interruption in hormone levels [24].

In the present study, we used a mouse model, in which mice were treated with acute myeloid leukemia (AML) cells and developed leukemia, to examine the direct effect of AML and chemotherapy (cytarabine)—alone and in combination—on sperm parameters and male fertility.

## 2. Results

### 2.1. Effect of AML Cells, Cytarabine, and the Combination of Both on Mice Survival

**Figure 1:**
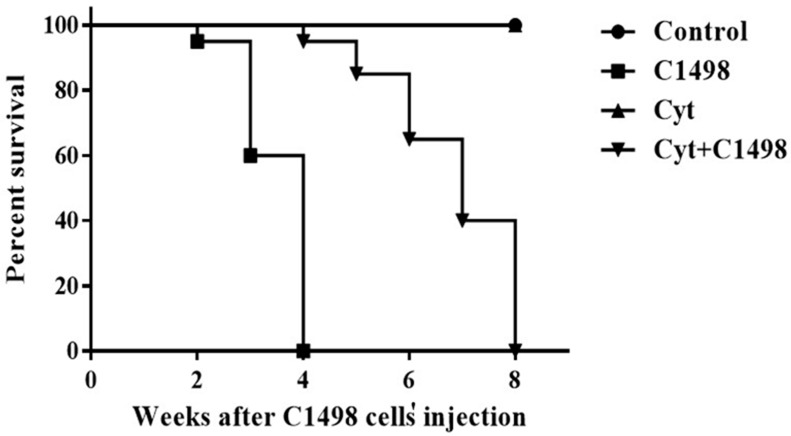
Effect of AML cells, cytarabine, and the combination of both on mice survival: Adult C57/black mice were injected with PBS (control), C1498 cells (C1498), cytarabine (Cyt), or with a combination of both (Cyt + C1498). The survival of mice was evaluated 2–8 weeks after injection (see the Methods) in intervals of one week. The results are representative of four independent experiments with 10 mice in each group.

Our results show that injection of AML cells (C1498) led to gradual death of the treated mice beginning two weeks after injection (95% survival). There was 60% survival after three weeks and 0% survival (100% death) after four weeks (Figure 1). Injection of the mice with PBS (control) or cytarabine (Cyt) did not affect survival (Figure 1). However, injection of cytarabine following AML injection (Cyt + C1498) led to an improvement in the number of mice that survived and the extension of the period of their survival. Mice started to die after four weeks of treatment (95% survival), and the percentage of survival was 80% after 5 weeks, 60% after 6 weeks, 40% after 7 weeks, and 0% after 8 weeks (Figure 1).

### 2.2. Effect of AML Cells, Cytarabine, and the Combination of Both on Sperm Concentration

**Figure 2:**
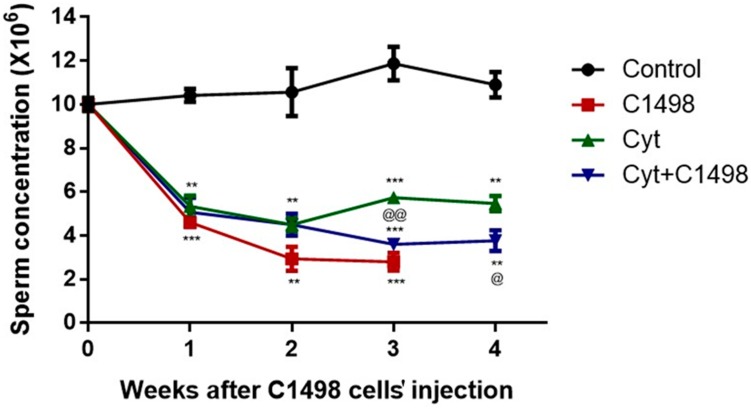
Effect of AML cells, cytarabine, and the combination of both on sperm concentration: Mice were treated as described in Figure 1. Sperm were extracted from the epididymis 1–4 weeks post-treatment. Sperm concentration was evaluated using a Makler counting chamber and determined according to WHO criteria. The results are representative of three independent experiments with 8–10 mice in each group. * Significant compared to control. @ Significant for Cyt + C1498 compared to Cyt. *, @ *p* < 0.05; **, @@ *p* < 0.01; ***, @@@ *p* < 0.001.

Our results showed a constant concentration of sperm in the control group mice within four weeks of the experiment. However, injection of AML cells (C1498) significantly decreased the sperm concentration after 1–3 weeks compared to the control (Figure 2). Furthermore, injection of cytarabine (Cyt), alone or in combination with C1498 (Cyt + C1498), led to a significant reduction in sperm concentration 1–4 weeks after the injection. This reduction was more significant compared to Cyt alone after three and four weeks post-treatment (Figure 2).

### 2.3. Effect of AML Cells, Cytarabine, and the Combination of Both on Sperm Motility

**Figure 3:**
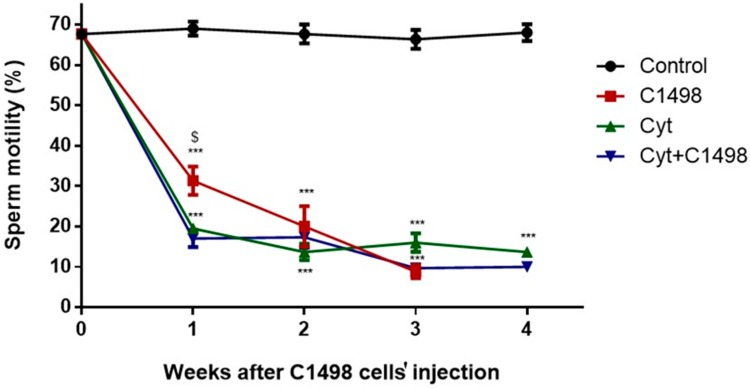
Effect of AML cells, cytarabine, and the combination of both on sperm motility: Mice were treated as described in Figure 1. Sperm were extracted from the epididymis 1–4 weeks post-treatment. Sperm motility/immotility was evaluated using a Makler counting chamber and determined as a percentage of total sperm according to WHO criteria. The results are representative of three independent experiments with 8–10 mice in each group. * Significant compared to control. $ Significant for Cyt + C1498 compared to C1498. *, $ *p* < 0.05; **, $$ *p* <0.01; ***, $$$ *p* < 0.001.

Our results show constant sperm motility in the control group mice within the four weeks of the experiment (Figure 3). However, injection of AML cells (C1498) alone, or cytarabine alone (Cyt), or in combination (Cyt + C1498) showed a significant decrease in the motility of the sperm after 1–3 weeks following C1498 and after 1–4 weeks following the other treatments compared to the control (Figure 3). The combination of C1498 and cytarabine (Cyt + C1498) led to a significant reduction in sperm motility compared to Cyt after one week of treatment (Figure 3).

### 2.4. Effect of AML Cells, Cytarabine, and the Combination of both on Sperm Morphology

**Figure 4:**
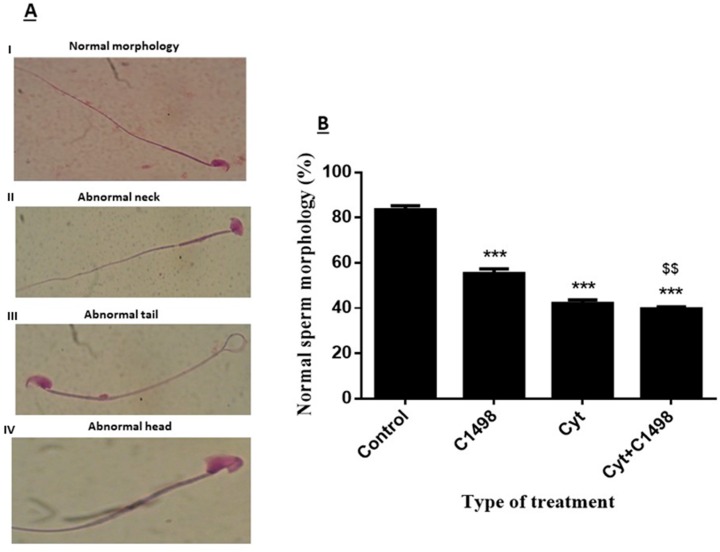
Effect of AML cells, cytarabine, and the combination of both on sperm morphology: Mice were treated as described in Figure 1. Sperm were extracted from the epididymis three weeks post-treatment. Sperm morphology was evaluated following staining with Diff-Quick stain (magnification of ×1000) (**A**). Cells were divided into different types of morphology: (I) normal morphology, (II) abnormal neck, (III) abnormal tail, (IV) abnormal head according to WHO criteria. The percentage of sperm with normal morphology was calculated (**B**). The results are representative of three independent experiment with three mice in each group. * Significant compared to control. $ Significant of Cyt + C1498 compared to C1498. $$ *p* < 0.01; *** *p* < 0.001.

Sperm with normal morphology were considered those with a normal head, neck, and tail (Figure 4A–I). However, abnormal sperm were considered to have different morphological abnormalities: abnormal neck (4A-II), abnormal tail (4A-III), and/or abnormal head (4A-IV). We found that in the control group, over 80% of the sperm had normal morphology after three weeks of treatment (control) (Figure 4B). However, three weeks following injection of the leukemic cells (C1498), there was a significant decrease in the percentage of sperm with normal morphology compared to the control (only 58% were normal) (Figure 4B). Three weeks after injection of cytarabine, only 45% of the sperm showed normal morphology. Injection of both leukemic cells and cytarabine (C1498 + Cyt) significantly decreased the normal morphology (40% normal) compared to the control (Figure 4B). This effect was more significant compared to injection of C1498 alone (Figure 4B).

### 2.5. Effect of AML Cells, Cytarabine, and the Combination of Both on Spontaneous Sperm Acrosome Reaction

**Figure 5:**
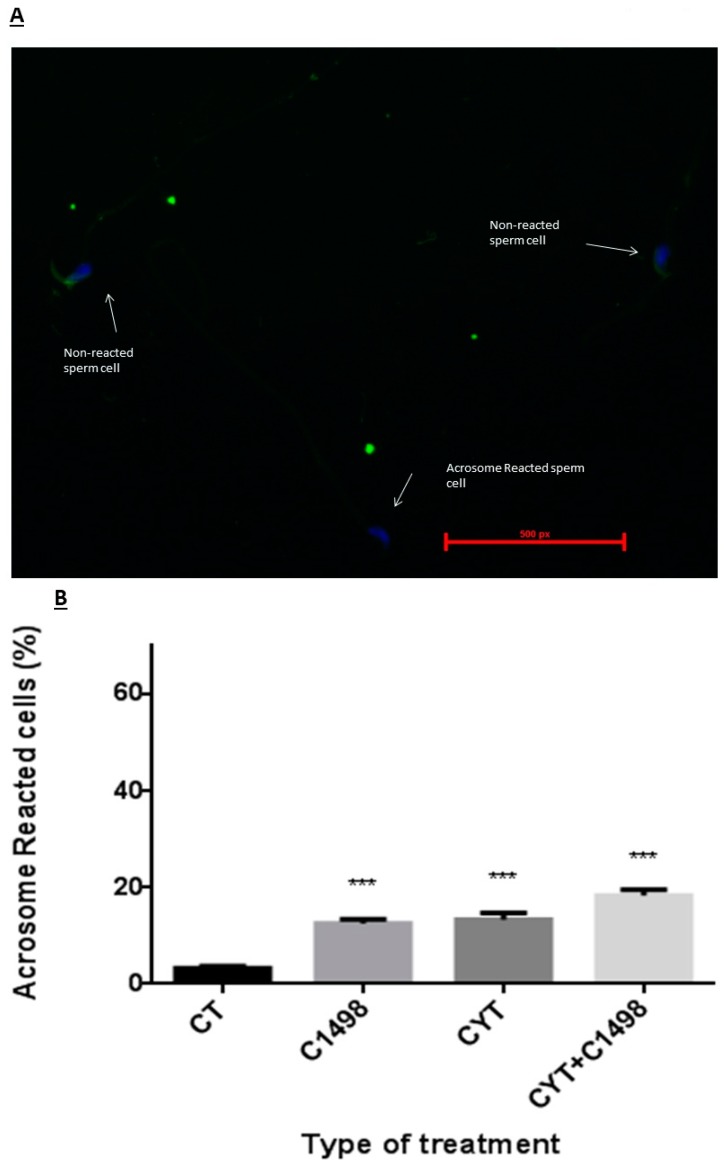
Effect of AML cells, cytarabine, and the combination of both compared to control (CT) on sperm spontaneous acrosome reaction: Mice were treated as described in Figure 1. Three weeks post-treatment, sperm were extracted from the epididymis and stained by fluorescein (FITC) staining (**A**). Acrosome reacted (without green staining) and non-reacted sperm (with green staining) were counted (A), and the percent of sperm that underwent spontaneous acrosome reaction was calculated (**B**). The results are representative of three independent experiment with four mice in each group. * Significant compared to control. *** *p* < 0.001.

In order to understand whether the fertility declines of the leukemic mice were related only to poor quality of sperm parameters (low concentration, low motility, and increase in abnormal morphology) or also due to the inability of the sperm to undergo the necessary processes of capacitation and acrosome reaction in order to fertilize the egg, we performed an acrosome reaction test (Figure 5A) and evaluated the percent of sperm cells that spontaneously underwent acrosome reaction following the different treatments compared to the control (Figure 5B). Our results showed that three weeks after C1498 or cytarabine (Cyt) injection, there was a significant increase in the percentage of sperm that underwent spontaneous acrosome reaction (Figure 5B). Three weeks after the injection of both C1498 and Cyt, there was a significant increase in the percentage of sperm with this reaction compared to the control, but it was not significant compared to the injection of C1498 or Cyt alone (Figure 5B).

### 2.6. Effect of AML Cells, Cytarabine, and the Combination of Both on Mouse Fertility Capacity and Number of Delivered Offspring

**Figure 6:**
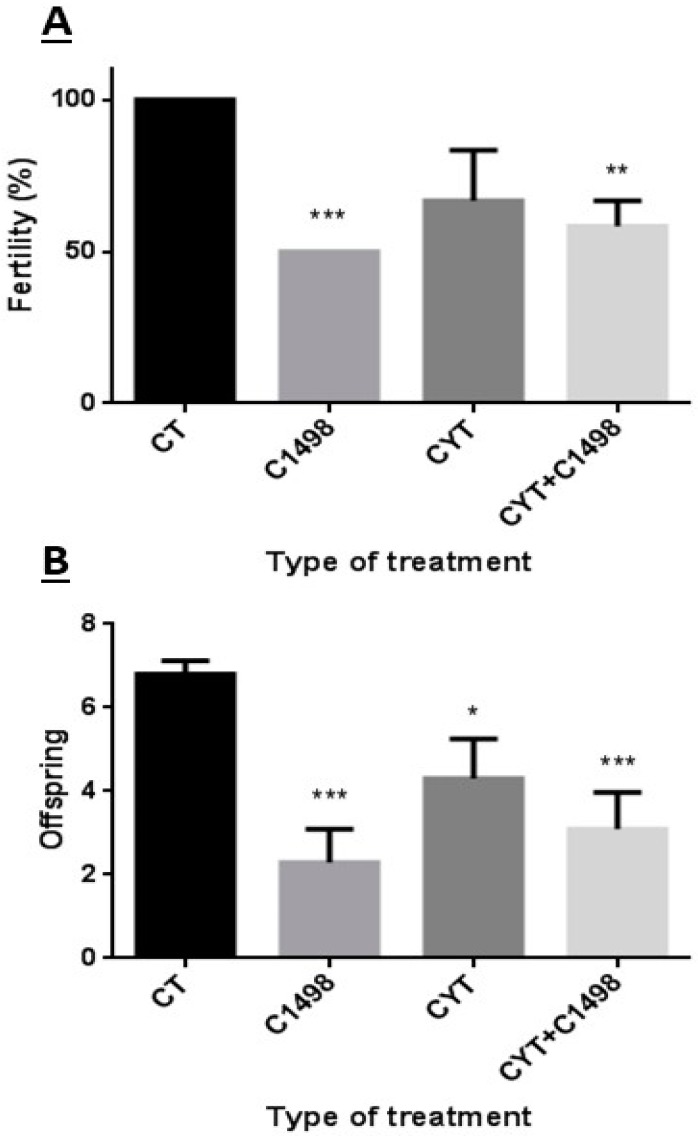
Effect of AML cells, cytarabine, and the combination of both on mice fertility and number of offspring: Mice were treated as described in Figure 1. Two weeks post-treatment, a single male from each group was mated with two females. After two weeks, the females were separated each to a single cage. The number of pregnant females (**A**) and number of offspring from each female were counted after five weeks (**B**). The results are representative of three independent experiments with four mice in each group. * Significant compared to control. * *p <* 0.05; ** *p* < 0.01; *** *p* < 0.001.

After we demonstrated that leukemia has an effect on sperm parameters, we examined whether there was an effect of the above treatment on male mouse fertility. Our results showed that three weeks after injection of C1498, male fertility was significantly reduced (50%) compared to the control (Figure 6A). However, even though injection of cytarabine (Cyt) reduced male fertility three weeks post-injection, this reduction was not significant compared to the control (Figure 6A). On the other hand, three weeks after injection of both C1498 and cytarabine (Cyt + C1498), male fertility was significantly reduced (60%) compared to the control (Figure 6A). In parallel to the reduction in male fertility following C1498 and cytarabine injection, we demonstrated a significant reduction also in the number of delivered offspring compared to the control (Figure 6B).

## 3. Discussion

In the present study, we showed that the AML-developed animal model led to the death of the mice within four weeks and that treatment of these mice with cytarabine chemotherapy extended their life to eight weeks (doubled their life). Using this model, we demonstrated that AML directly affected the different sperm parameters (concentration, motility, morphology). These results are in harmony with previous studies in cancer and AML patients [14,15,16,17,18,19,20,21,22,23,24,25,26,27] and demonstrate, for the first time, a direct effect of AML disease on mouse male sperm parameters similar to AML patients. In addition, we showed a direct effect of AML disease on mouse male infertility. We also demonstrated, for the first time, that reduction in male fertility of AML mice could be related to an increase in spontaneous acrosome reaction of the sperm, in addition to a reduction in other sperm parameters. Reduction in sperm concentration and an increase in abnormal morphology of sperm following AML may indicate an effect of the disease on spermatogenesis. This effect could be related to changes in hormones that affect spermatogenesis such as gonadotropins and testosterone [28,29,30,31,32,33,34]. Furthermore, AML could affect systemic inflammatory cytokines (growth factors) and/or testicular (autocrine/paracrine) factors that may affect normal spermatogenesis (affect the proliferation and differentiation of SSCs, and even their apoptosis) and lead to male subfertility/infertility [23,24,25,26,27,35,36,37,38,39,40]. The effect of AML on sperm motility and spontaneous acrosome reaction may indicate a direct effect of the disease on the normal functionality of the epididymis. It should be noted that the effect of AML on the examined sperm parameters was similar or even more potent (on sperm concentration) than cytarabine. Furthermore, the effect of AML on the fertility capacity and spontaneous acrosome reaction was more pronounced than cytarabine. On the other hand, the combination of AML and cytarabine significantly increased the normal morphology compared to cytarabine, but did not show any pronounced effect on sperm concentration and motility compared to each of them alone. These results may suggest that AML and cytarabine differently affect the various stages of spermatogenesis. Whereas they similarly affect sperm generation, they differently affect sperm morphology. Therefore, they are more effective (together) at impairing normal sperm morphology. Together, they also did not show more effectiveness on spontaneous acrosome reaction, which may indicate a similar level of effect on sperm functionality in the epididymis. 

In conclusion, our results support previous findings that AML disease impairs sperm parameters, and we show, for the first time, its role in increasing spontaneous acrosome reaction and its possible involvement in decreasing male fertility capacity. We also suggest possible different roles for AML and cytarabine in the generation of sperm, in spermiogenesis in the testis, and in sperm motility and acrosome reaction in the epididymis.

Thus, AML alone can affect male infertility, and the addition of chemotherapy will increase this pathology. Understanding the mechanisms of the action of AML and chemotherapy may lead to the development of future therapeutic strategies for male infertility in cancer patients. 

## 4. Materials and Methods

### 4.1. Animals

This study was performed in accordance with the Guiding Principles for the Care and Use of Research Animals Promulgated by the Society for the Study of Reproduction. It was confirmed by the Ben-Gurion University Ethics Committee for Animal Use in Research (IL-70-11-2016). Six-week-old C57/black mice were purchased from Envigo Laboratories, Jerusalem, Israel. 

### 4.2. C1498/Cell-Line Preparation and Injection

Murine C1498 (TIB-49) AML cells were purchased from American Type Culture Collection (Rockville, MD, USA). They were cultured in RPMI 1640 medium supplemented with 10% FBS, penicillin (100 U/mL), streptomycin (0.1 mg/mL), and 10 mM HEPES (pH = 7.4) in a humidified atmosphere of 95% air and 5% CO_2_, at 37 °C. Then, 10^5^ cells/100 µL were injected intravenously per mouse.

### 4.3. Cytarabine Preparation and Injection

Cytarabine (powder) was purchased from Sigma (Sigma-Aldrich Israel Ltd., Rehovot, Israel) and dissolved in sterile PBS. Mouse weight was evaluated before injection, with 100 µL of Cyt (3 mg/kg) injected intraperitoneal into each mouse. The injections were performed 24 h after the injection of C1498 cells, 3 times every 12 h (according to Lin, J.M, et al., [41]; with adaptation). As a control, mice were injected with 100 µL of sterile PBS.

### 4.4. Sperm Extraction from Epididymis

Mice were sacrificed at different time points after C1498 cell injection, and the epididymis was removed. Sperm cells were extracted from the tail of the epididymis by squeezing in a Petri dish plate. Cells were collected in a small tube.

### 4.5. Sperm Concentration and Motility Measurement

Collected sperm (as described above) were examined for concentration and motility using a Makler counting chamber. For sperm concentration determination, 10 µL of each sample was transferred to a chamber, and cells were counted at a total microscope magnification of ×400. For sperm motility evaluation, only motile cells were counted without differentiation of motility type.

### 4.6. Morphology Measurement

Semen smears were made and stained by use of Quik Stain (Biological Industries, Cromwell, CT, USA). Evaluation of sperm morphology was performed using an upright microscope under a magnification of ×1000 (using immersion oil). This was performed according to the WHO criteria [42].

### 4.7. Assessment of Sperm Acrosome Reaction

The percentage of acrosome-reacted sperm was determined microscopically on air-dried sperm smears using fluorescein conjugated peanut agglutinin (FITC-PNA) (Sigma-Aldrich Israel Ltd., Rehovot, Israel). An aliquot of sperm cells was smeared on a glass slide and allowed to air dry. The sperm were then permeabilized by methanol for 15 min at room temperature, washed three times at 5-min intervals with PBS, and air dried. Thereafter, the slides were incubated with FITC (50 µg/mL in PBS) for 30 min, washed twice with H_2_O at 5-min intervals, and mounted with DAPI (Santa Cruz Biotechnology, Santa Cruz, CA, USA) to stain cell nuclei. For each experiment, at least 200 cells per slide (on duplicate slides) were evaluated (total of 400 cells for one experiment). Cells with green staining over the acrosomal cap were considered acrosome intact; those with equatorial green staining or no staining were considered acrosome reacted.

### 4.8. Fertility Capacity Test

Male mice were injected with PBS (control), C1498 cells, cytarabine alone, or a combination of both cytarabine and C1498. Two weeks after treatment, a single male from each treatment was mated with 2 females (8 weeks old). After two weeks, the females were each separated in different cages. The number of pregnant females and the number of offspring for each female was examined after 4–5 weeks of separation.
